# Efficient production of protein complexes in mammalian cells using a poxvirus vector

**DOI:** 10.1371/journal.pone.0279038

**Published:** 2022-12-15

**Authors:** Robert Drillien, Karine Pradeau-Aubreton, Julien Batisse, Joëlle Mezher, Emma Schenckbecher, Justine Marguin, Eric Ennifar, Marc Ruff

**Affiliations:** 1 Department of Integrative Structural Biology, IGBMC, University of Strasbourg, CNRS UMR 7104, INSERM U964, Illkirch, France; 2 Structure et Dynamique des Machines Biomoléculaires, Institut de Biologie Moléculaire et Cellulaire, UPR 9002 CNRS/Université de Strasbourg, Strasbourg, France; University of Rochester School of Medicine and Dentistry, UNITED STATES

## Abstract

The production of full length, biologically active proteins in mammalian cells is critical for a wide variety of purposes ranging from structural studies to preparation of subunit vaccines. Prior research has shown that Modified vaccinia virus Ankara encoding the bacteriophage T7 RNA polymerase (MVA-T7) is particularly suitable for high level expression of proteins upon infection of mammalian cells. The expression system is safe for users and 10–50 mg of full length, biologically active proteins may be obtained in their native state, from a few litres of infected cell cultures. Here we report further improvements which allow an increase in the ease and speed of recombinant virus isolation, the scale-up of protein production and the simultaneous synthesis of several polypeptides belonging to a protein complex using a single virus vector. Isolation of MVA-T7 viruses encoding foreign proteins was simplified by combining positive selection for virus recombinants and negative selection against parental virus, a process which eliminated the need for tedious plaque purification. Scale-up of protein production was achieved by infecting a BHK 21 suspension cell line and inducing protein expression with previously infected cells instead of virus, thus saving time and effort in handling virus stocks. Protein complexes were produced from infected cells by concatenating the Tobacco Etch Virus (TEV) N1A protease sequence with each of the genes of the complex into a single ORF, each gene being separated from the other by twin TEV protease cleavage sites. We report the application of these methods to the production of a complex formed on the one hand between the HIV-1 integrase and its cell partner LEDGF and on the other between the HIV-1 VIF protein and its cell partners APOBEC3G, CBFβ, Elo B and Elo C. The strategies developed in this study should be valuable for the overexpression and subsequent purification of numerous protein complexes.

## Introduction

Overexpression of proteins as well as protein complexes in higher eukaryotic cells is performed for a variety of applications in research, diagnostics and therapeutics. Such strategies are particularly valuable for proteins from higher eukaryotes that require their natural environment for folding, post-translational processing and optimal solubility. Many alternative strategies have been developed, each with its own advantages and disadvantages. Virus vectors are particularly suitable because they naturally amplify their genomes in infected cells thus providing multiple copies of the genes to be expressed. Furthermore, viruses are readily propagated in a single host cell line thereby avoiding the need to handle distinct cell lines for each gene and viruses are less cumbersome to store and recover from storage than cell lines. As compared to transient expression upon nucleic acid transfection, a powerful method for protein production, virus infection more readily allows expression in entire cell cultures without the need for transfection agents or large amounts of nucleic acid. Thus our focus has been to take advantage of the ability of a member of the Poxvirus family, namely Modified Vaccinia Ankara engineered to encode the bacteriophage T7 RNA polymerase (MVA-T7), to amplify its genome within infected hamster cells and induce expression of any gene that has been integrated into the viral genome downstream of a bacteriophage T7 promoter [1,2]. Gene expression from this viral vector is tightly regulated by an *E*. *coli* lac operator and lac repressor switch under the control of the inducer **isopropyl β-D-1-thiogalactopyranoside** (IPTG) [3,4]. Using this strategy our laboratory has overexpressed and purified several full length cellular and viral proteins for functional and structural studies [5–8]. Despite its efficiency, the current MVA expression system still has a number of drawbacks such as the time required to isolate virus recombinants, the difficulty to scale up protein production and to co-express multiple proteins belonging to a protein complex upon infection with a single virus. Here we report strategies to address each of these issues.

## Material and methods

### Cells and their propagation

Adherent Syrian baby hamster kidney cells (BHK 21) and suspension-adapted BHK 21 C13-2P cells (European Collection of Cell Cultures, catalogue number 84111301) were both cultured in G-MEM supplemented with 10% fetal calf serum, 1,5 g/L bacto tryptose phosphate and 40 mg/L gentamicin.

### Plasmids for the construction of virus recombinants

The pKoom plasmid used to generate the MVA-Koom virus was constructed from pGP-GNR, pRSET-mCherry and pVote2 kindly provided by James Jancovich, Jean-Marie Garnier and Bernard Moss respectively (S1 Fig). The BioBrick strategy was set up as follows. Twin TEV cleavage sites, purification tags (10His, Flag10His, 10HisFlag, TwinStrep, FlagTwinStrep and TwinStrepFlag) fused to a P3C cleavage site (S2A Fig), and primers were purchased from Sigma. Synthetic DNA encoding the HIV-1 codon modified IN, LEDGF and YFP were purchased from GeneArt or GeneScript respectively. PCR was used to amplify TEV, APOBEC3G, CBFβ, EloB, EloC, HIV-1 Vif and IN (pNL4.3) with the primers listed (S2B Fig). Translational stop signals were removed from all ORFs and replaced with a series of degenerate codons for twin TEV cleavage sites (S2C Fig) except the YFP stop codon. All ORFs were flanked at their 5’ end by EcoRI and XbaI sites and at their 3’ end by SpeI and PstI sites (S2D Fig) in order to carry out a reiterative BioBrick cloning strategy and assemble a polyprotein coding sequence [9]. Briefly, as schematically represented in S3 Fig, ORFs were prepared with EcoRI and PstI digestion and inserted into pUC19 that was digested with EcoRI and PstI, to generate a pUC_BB plasmid. Each pUC_BB was further linearised with SpeI and PstI to add a downstream BioBrick generated by XbaI and PstI restriction, or linearized with EcoRI and XbaI to add an upstream Biobrick generated by EcoRI and SpeI restriction. The polyprotein coding sequences were then removed from the pUC_BB constructs by XbaI and PstI digestion and inserted into the pENTR_BB-TEV-YFP digested with SpeI and PstI. Finally, the pENTR_BB plasmids were recombined with the pVote0GW plasmid using Gateway recombination to generate a plasmid for insertion of the polyprotein sequences into the MVA genome as previously described [2].

### Selection and amplification of virus recombinants

To isolate the MVA-Koom virus, 10^6^ adherent BHK 21 cells were infected with MVA-T7 (an MVA virus encoding T7 RNA polymerase and GFP [2] at about 2 plaque forming units per cell (PFU/cell) and transfected in OptiMEM medium with 1 μg pKoom plasmid DNA using Lipofectamine 2000 (Thermo Fisher Scientific). Cells were frozen at -20°C one day later and subsequently freeze-thawed several times to release virus. MVA-Koom was selected by serial passage in the presence of 1 mg/ml G418 and visualization of mCherry positive plaques under a fluorescence microscope. Virus recombinants encoding genes under the control of the T7 promoter were generated as outlined in S4 Fig. Briefly, 10^6^ adherent BHK 21 cells were infected with MVA-Koom at about 2 PFU/cell and transfected as above. Cells were frozen at -20°C one day later and subsequently freeze-thawed several times to release virus. Released virus was diluted and used to infect fresh BHK 21 monolayers in the presence of 25 μg/ml mycophenolic acid, 15 μg/ml hypoxanthine and 250 μg/ml xanthine (MPA medium) and/or 100 ng/ml coumermycin. Two days later, cell cultures were checked for green fluorescence due to GFP expression and red fluorescence due to mCherry expression using a fluorescence imaging system. This process was repeated several times in the presence MPA and coumermycin, until all viral plaques displayed green fluorescence and no red fluorescence. At the end of the selection cycles, virus was amplified on BHK 21 cells in the absence of any selection pressure. Final virus stocks were prepared from infected cells displaying GFP expression within the entire cell culture as well as an overall cytopathic effect visible under a light microscope. Infected cell cultures were submitted to three cycles of freeze-thawing at -20° then centrifuged at 2000 g for 5 minutes. The supernatants were recovered, frozen at -20°C and used as crude virus stocks for protein expression experiments.

### Protein production and purification

Medium scale protein production was carried out in adherent BHK 21 cells grown in 175 cm^2^ plastic bottles or in suspension BHK 21 C13-2P cells grown up to 1–1.5 10^6^ cells/ml in cylindrical flasks submitted to orbital shaking. In the case of adherent cells, cell culture medium was removed and crude virus stocks were added to the cultures at about 2 PFU/cell for one hour incubation at 37°C. At this time, fresh medium containing 5–10% serum and 1 mM IPTG were added and incubation continued at 37° C for 24 hours. Infected cells were pelleted at 2000 g for 5 minutes, suspended in PBS and pelleted again at 2000 g for 5 minutes. Finally, the cell pellets were stored at -80° until their use for protein analysis or purification. Glutathion-S transferase (GST) tagged proteins were purified from infected cell pellets using a batch resin method (Glutathione-Sepharose-4B) and a protocol provided by GE Healthcare.

For large scale production of proteins, suspension BHK 21 C13-2P cells (1–1.5 cells/ml) were infected as above with about 0.1 PFU/cell in 5 liter cylindrical flasks containing 1.2 liters of cell culture medium. No IPTG was added to the cultures to allow amplification of the virus titer. Two days later, infected cells were mixed with uninfected cells at a 1:10 ratio in six 5 liter cylindrical flasks containing 1.5 to 2 liters of cell culture at 1–1.5 10^6^ cells/ml. IPTG (1 mM final) was added at the time of cell mixture. Incubation at 37°C was pursued for another 24 hours. Cells were then pelleted and washed as above and the pellets stored at -80°C until use.

For purification of Twin-Strep tagged proteins (Vif/APOBEC complex), cell pellets were lysed by sonication in a buffer containing 50 mM Tris-HCl (pH8), 500 mM NaCl, 1mM EDTA, 10 mM CaCl_2_, 1mM MgCl_2_, 7 mM β mercaptoethanol, 0,5% tween 20 and a protease inhibitor cocktail. The lysates were then treated for one hour with RNAse and DNAse at 4°C. Insoluble proteins were removed by ultracentrifugation at 100,000 g and the supernatants obtained were filtered through 5μm and 0.2 μm cellulose membranes. Tagged proteins were then retained on a 1ml Strep Tactin column, washed in three successive steps (1 M NaCl, 1 mM EDTA, 50 mM Tris-HCl pH 8 then 150 mM NaCl, 1 mM EDTA, 100 mM Tris-HCl pH8 then 10 mM NaCl, 50 mM Tris-HCl pH8, 5% glycerol, 10 μM ZnCl2 and eluted with 5 mM desthiobiotin in 50 mM Tris-HCl pH 8, 10mM NaCl, 10 μM ZnCl2, 5% glycerol. Peak fractions were collected, pooled together, concentrated on a 100 kDa ultracentrifugal filter unit by centrifugation at 4000 rpm for 5 min and analyzed by SDS PAGE and Coomassie staining.

For large scale purification of His-tagged IN/LEDGF complex, cell pellets were suspended in buffer A (50 mM Hepes pH 7.5, 400 mM NaCl, 2 mM MgCl2, 2 mM β mercaptoethanol, 1 tablet of a protease inhibitor cocktail (Roche)) containing 10 mM imidazole at a ratio of 5ml buffer for 1 g cell pellet. Cell suspensions were sonicated 15 minutes with a 2 seconds on/off cycle. The lysed cells were then centrifuged at 100,000 g for one hour and the supernatants recovered and filtered through a 5 μm cellulose acetate filter. The soluble proteins were then run through a 5 ml HisTrap excel Ni Sepharose column equilibrated with buffer A containing 10 mM imidazole. A 3-step wash was then performed with 10 column volumes of the same buffer (A with 10 mM Imidazole), then buffer A with 20 mM imidazole and buffer A with 40 mM imidazole. Finally, elution of His-tagged proteins was carried out with an imidazole gradient from 40 mM to 500 mM. Peak fractions were collected and analyzed by SDS PAGE and Coomassie staining. Fractions of interest were then pooled together and concentrated to 5 ml on a 100 kDa ultracentrifugal filter unit by centrifugation at 4000 rpm for 10 min. Proteins were then purified by size exclusion on a Hi Load 16/60 superdex-200 column (GE Healthcare) pre-equilibrated in buffer A. Peak fractions were collected and analyzed by SDS PAGE and Coomassie staining.

### Measurement of cytidine deaminase and integrase activities

The cytidine deaminase activity of APOBEC3G alone or in a multi-protein complex was determined following a previously described method with a few modifications [10]. Briefly, 40 μM of the 39 base pair oligonucleotide 5’ AAAGAGAAAGAGAAACCCAAAGAGGAAAGGTGAGGAGGA 3’, where the third C in the CCCA motif may be deaminated, was incubated in buffer A (20 mM Tris-HCl pH 7.5, 30 mM NaCl, 10 μM MgCl_2_ and 5 mM DTT) in the presence of infected cell lysates for two hours at 37°C. DNA was then extracted with phenol-chloroform, ethanol precipitated, solubilized in buffer A and treated with 10 units uracil glycosylase for two hours at 37°C to remove the uracils created by cytidine deaminase activity. Finally, alkaline hydrolysis of the abasic oligonucleotide for 20 min at 95°C in 0.2 M NaOH cut the abasic 39 oligonucleotides into 17 and 21 long oligonucleotides which were separated on an anion exchange column (Nucleopac PA-100 form Dionex).

The IN 3’processing activity was monitored by fluorescence anisotropy as described previously [11]. The reaction was done in 96 well-plates. One well contained 100 μL of reaction mix composed of 25 mM BisTris pH 6.5, 10 mM MgCl2, 5 mM DTT, 50 nM DNA and 200 nM protein complex. The DNA substrate was a 40 base pair double strand DNA (5’-GACTACGGTTCAAGTCAGCGTGTGGAAAATCTCTAGCAGT-3’), mimicking the U5 end of HIV-1 DNA and 3’ modified by 6-fluorescein. After homogenization, 25μL of paraffin oil was added on the top of the well to avoid evaporation. The reaction was monitored for 6 hours at 37°C. A decrease in fluorescence anisotropy as measured on a PHERAstarPlus (BMGLab) spectrophotofluorimeter with an excitation polarized wavelength of 470 nm indicated enzymatic activity.

## Results

### Streamlining recombinant virus isolation

In studies conducted previously we inserted foreign genes to be expressed into the MVA-T7 genome by transfecting infected cells with a plasmid containing the gene downstream of a T7 promoter and adjacent to the gene encoding the *E*. *coli* xanthine-guanine phosphoribosyl transferase (GPT). *In vivo* recombination between the MVA-T7 genome and the plasmid resulted in the simultaneous insertion of both genes into the viral genome and allowed selection of virus recombinants on the basis of their ability to multiply in medium containing mycophenolic acid, xanthine and hypoxanthine [12], hereafter referred to as MPA medium. Despite this selective pressure, multiple steps of plaque purification were necessary to isolate a clonal population of recombinant virus devoid of parental contaminants. To speed up the process of recombinant virus isolation and simplify it we decided to introduce a negative selection pressure against the parental virus in addition to the positive selection pressure for virus recombinants provided by the GPT gene. For this purpose we added to the parental viral genome (MVA-T7) the fusion gene GyrB-PKR comprising residues 1–220 of the *E*.*coli* gyrase B fused to the kinase domain (residues 258–551) of human dsRNA dependent protein kinase (PKR) following a procedure devised by White et al. [13] Expression of this GyrB-PKR fusion gene is inhibitory for viral multiplication only in the presence of coumermycin which induces dimerization of the fusion protein, activation of PKR and inhibition of protein synthesis [13,14]. The addition of the GyrB-PKR fusion gene to the viral genome was ensured by simultaneous insertion of another fusion gene (mCherry-NeoR) which confers both red fluorescence and resistance to neomycin as well as its analog G418 [15]. Both the GyrB-PKR and mCherry-NeoR fusion genes were inserted together at the viral hemagglutinin locus which is routinely targeted for insertion of foreign genes. We thus obtained an MVA-T7 vector named MVA-Koom which multiplied well in BHK 21 cells under standard conditions but was inhibited when coumermycin was added to the medium as previously demonstrated by White et al for the vaccinia virus WR strain [13].

**Fig 1:**
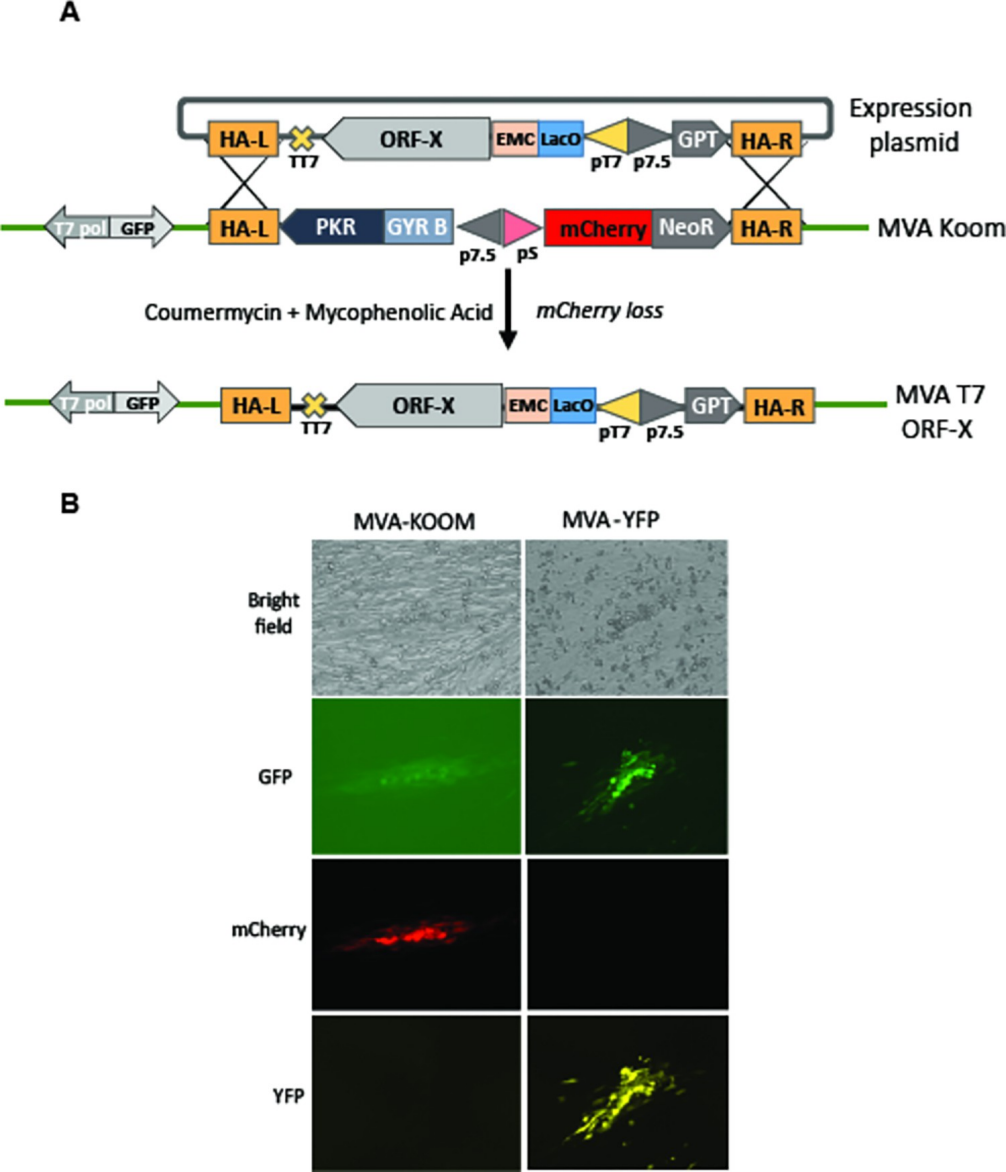
Construction of virus recombinants by combined positive and negative selection. A. A vaccinia virus expression plasmid encoding an ORF of interest (ORF-X) is cloned downstream of a bacteriophage T7 promoter (pT7) under the additional transcriptional control of a Lac operator (LacO), an encephalomyocarditis (EMC) virus leader sequence and a bacteriophage transcriptional stop sequence (TT7). A positive selection marker (GPT behind a 7.5 early/late vaccinia virus promoter) is included on the plasmid. Sequences on the left and right sides of the vaccinia virus hemagglutinin gene (HA-L and HA-R) are used in vivo to recombine with the MVA-Koom virus which encodes T7 RNA polymerase, GFP as well as the GYR-PKR and the mCherry-NeoR fusion genes. Replacement of the fusion genes by OFF-X is selected by the ability of the recombinant virus to multiply in the presence of MPA and coumermycin resulting in loss of mCherry expression. Note that the sequences are represented according to the standard orientation of the virus genome. B. Analysis of recombinant virus plaques by light and epifluorescence microscopy. BHK 21 cells were infected in the presence of IPTG with MVA-Koom or an MVA-T7 recombinant virus derived from MVA-Koom by insertion of a polyprotein sequence encoding YFP at its 3’ end (see Fig 4). One representative virus plaque for each infection was observed with an EVOS Cell Imaging System using from top to bottom a white light cube (top panel), a GFP light cube (470/22, 510/42), a YFP light cube 500/24, 524/27 or an RFP light cube (531/40, 593/40).

Virus recombinants encoding a variety of genes under the control of a T7 promoter were generated by infecting cells with MVA-Koom and transfecting the same cells with plasmids encoding the genes to be expressed under the control of a T7 promoter flanked by the recombination arms of the hemagglutinin locus (Fig 1A). To isolate the rare recombination events, a first round of selective pressure was carried out in the presence of MPA medium. Both red (mCherry positive) and green (GFP positive) plaques were visible under a fluorescent microscope as the MVA Koom virus encodes both mCherry and GFP (adjacent to the gene encoding the T7 RNA polymerase). Virus from this first round of selection was then passaged twice on BHK 21 cells in the presence of MPA medium containing coumermycin. Under these conditions, plaques displaying green fluorescence but no red fluorescence were observed indicating that negative selection against the parental virus had occurred because a new gene had replaced the mCherry-NeoR and GyrB-PKR fusion genes. Under the microscope, virus plaques formed by the MVA-Koom parental virus displayed weak green fluorescence and strong red fluorescence in the absence of the inducer IPTG or in its presence (Fig 1B). Virus plaques formed by a recombinant virus encoding YFP downstream of a T7 promoter, isolated following the combined positive and negative selection method, no longer displayed red fluorescence but now displayed yellow fluorescence due to induction of the T7 RNA polymerase by IPTG and strong green fluorescence due to an overlap of the YFP signal in the wavelength selected by the GFP filter. In the absence of IPTG induction, no yellow fluorescence or strong green fluorescence was observed. This isolation strategy, which involves no virus plaque picking, has been used routinely to obtain dozens of virus recombinants with no failures although as discussed below with variable levels of expression of individual proteins.

### Protein production in suspension cell cultures

**Fig 2:**
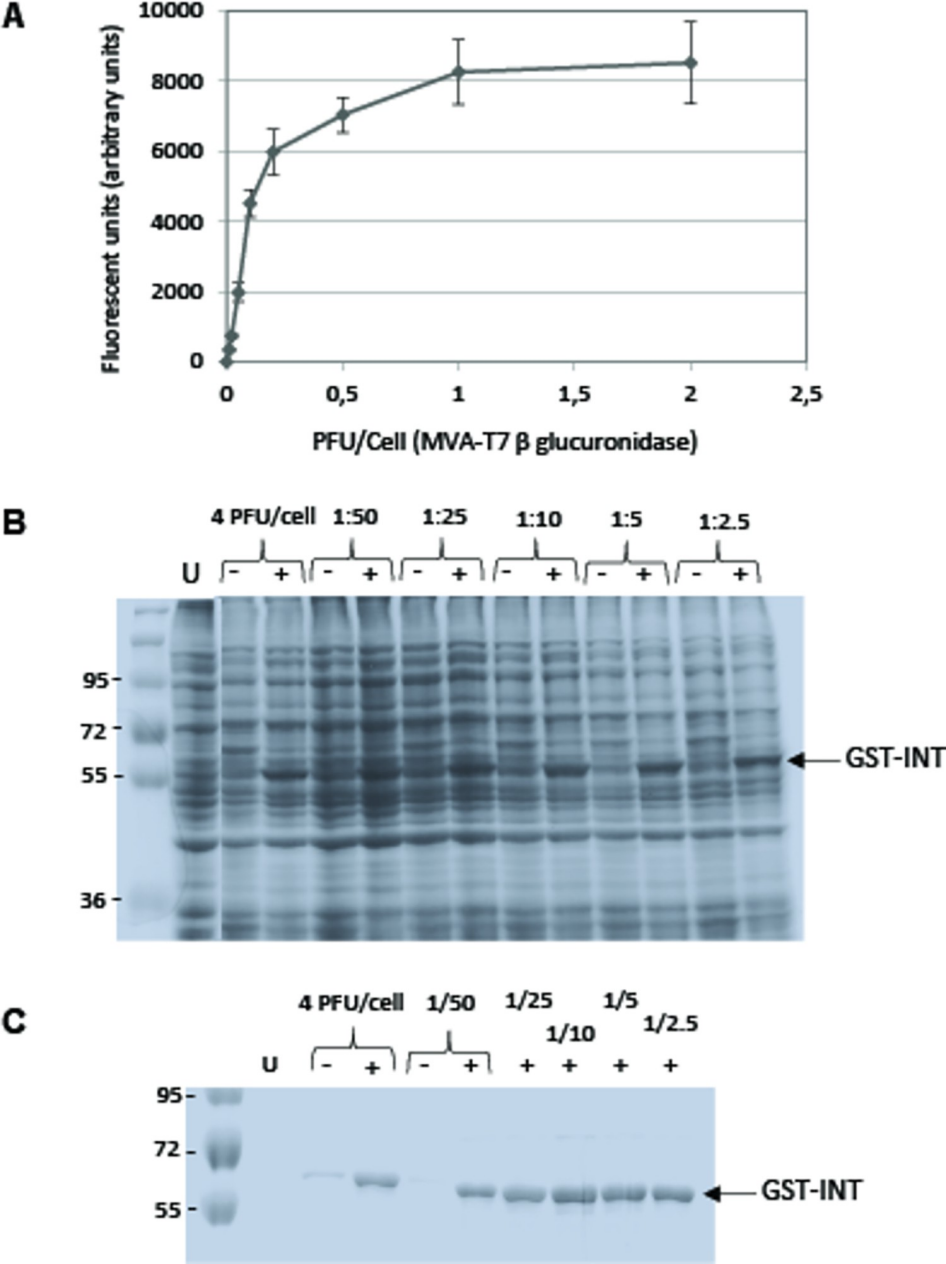
Protein production in suspension cell cultures. A. BHK 21 suspension cells (1.2 10^6^ cells/ml) were infected in 50 ml cell culture medium with MVA-T7 encoding β glucuronidase at increasing MOIs. After 24 hours infection cells were pelleted and enzymatic activity determined in duplicate. B. BHK 21 suspension cells (10^6^ cells/ml) were infected in a 100 ml cell culture medium with an MVA-T7 virus encoding a GST-tagged HIV-1 integrase at 0.1 PFU/cell. After 48 hours infection in the absence of IPTG, 1 ml, 2 ml, 5 ml, 10 ml or 20 ml of infected cells were added to 50 ml of uninfected cells (10^6^ cells/ml). Cell culture medium was added so that the culture volumes were identical in all samples and protein expression was induced by the simultaneous addition of IPTG. Control infections were conducted with 4PFU/cell of the MVA-T7 virus encoding GST-integrase in the presence or absence of IPTG. 24 hours later 1.5ml of the infected cell cultures were recovered for analysis of total protein by PAGE. U uninfected cells.–and + indicate the presence or absence of IPTG in the infected cell cultures. C. 45 ml samples of the infected BHK 21 suspension cultures in panel B were pelleted, sonicated and the GST-integrase purified and examined by PAGE. U uninfected cells.–and + indicates the absence or presence of IPTG in the infected cell cultures.

**Fig 3:**
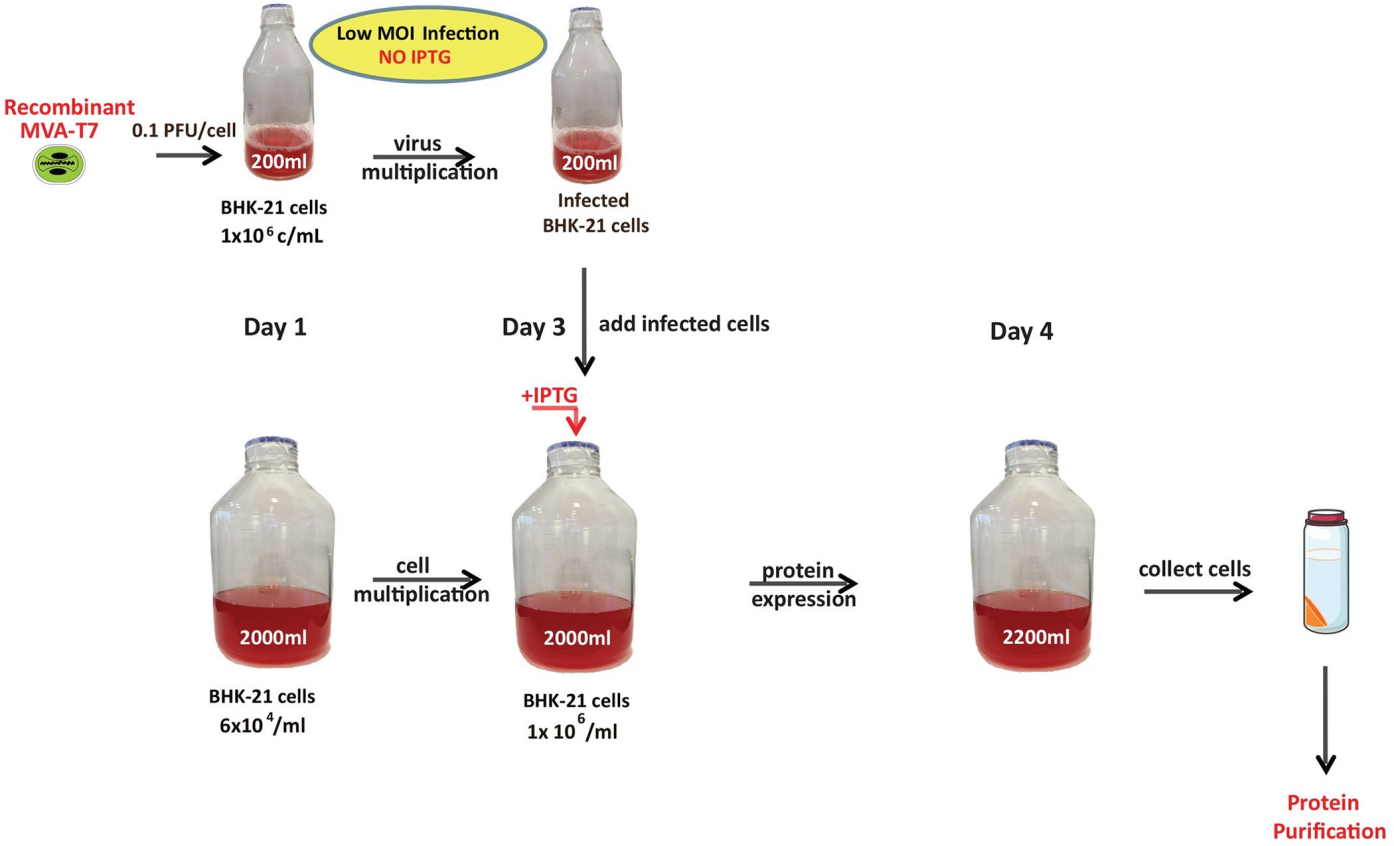
Overview of mammalian protein expression in infected cells. Schematic illustration of the mammalian expression system. The top part of the figure depicts amplification of virus in a preproduction setting in the absence of inducer (IPTG). The bottom part depicts multiplication of uninfected cells for two days followed by addition of infected cells to uninfected cell cultures. Inducer is added upon cell mixing and one day latter infected cells are recovered and purification of protein complexes can be carried out.

In prior studies we have used adherent BHK 21 cells for protein production after infection with MVA recombinants [1,2,5]. However, adherent cells are not practical for handling large cell volumes particularly for expression and recovery of proteins that remain intracellular as is the case for the gene products we have expressed so far. Adherent cells must be treated with trypsin at each passage when they reach confluence and their recovery after infection requires scraping which is a time-consuming process that can damage cells. We therefore sought to use a suspension cell line (BHK 21-C13-2P), derived from the original BHK 21 cell line, which we cultivated in cylindrical flasks of various sizes and submitted to orbital shaking under standard cell culture conditions (5% CO_2_ at 37°C). Initial experiments demonstrated that the amount of protein produced in suspension cells on a per cell basis was about 2.5 fold lower than the amount produced in adherent cells (S1 Table). However, the suspension cells could be grown to higher densities so that the same volume of cells from either type of culture led to equivalent protein yields. Since suspension cells were also easier to handle in large volumes we chose to further explore the potential of this cell culture system. To determine the optimal virus concentration for expression over a 24-hour period, suspension cell cultures were infected in the presence of the inducer IPTG with an MVA-T7 recombinant virus encoding β glucuronidase at a range of multiplicities of infection (MOI). Protein yields were highest when the MOI was 1 or 2 PFU per cell (Fig 2A). The MOI required for optimal protein production being relatively high and the viral stocks that could be produced having at best a titer in the range of 10^7^ PFU/ml implied that it would be necessary to produce large amounts of virus stock to induce protein expression in several liters of cell culture as is often necessary to obtain sufficient amounts for downstream analysis. We considered that a virus saving method previously devised for protein expression with Baculovirus recombinants [16] could be useful. In this procedure insect cells are infected for protein production with previously infected cells rather than virus. To assay this method, suspension cells were infected with an MVA recombinant encoding a model protein (GST-tagged HIV-1 integrase) at a low MOI (0.1 PFU/cell) in the absence of any inducer and virus amplification allowed to proceed for two days. At this point the cell culture was checked for successful infection by ensuring that all cells displayed GFP fluorescence. Infected cells were then mixed with a suspension culture of uninfected cells at various ratios from 1:50 up to 1:2.5 (Fig 2B). A control infection with virus instead of infected cells showed that the GST-integrase was detected by SDS-PAGE of the total cell extract in the presence of the IPTG inducer but not in its absence. Interestingly, cells that had been mixed with infected cells produced roughly as much GST-integrase as cells directly infected with virus at all cell ratios indicating that the virus saving procedure was promising. To more clearly determine the relative amount of GST-integrase produced we purified GST-integrase using a glutathione resin and compared the relative protein yields. We could confirm under these conditions that the GST-integrase was produced in the mixed cell cultures at levels comparable to those in the infected cell culture (Fig 2C). A ratio of 1 infected cell per 10 uninfected cells was sufficient to produce the highest protein yield so this ratio has been routinely used in numerous protein production runs involving up to 24 liters of infected cell culture. A schematic representation of the overall production procedure is shown in Fig 3.

### The polyprotein precursor strategy applied to two HIV complexes

**Fig 4:**
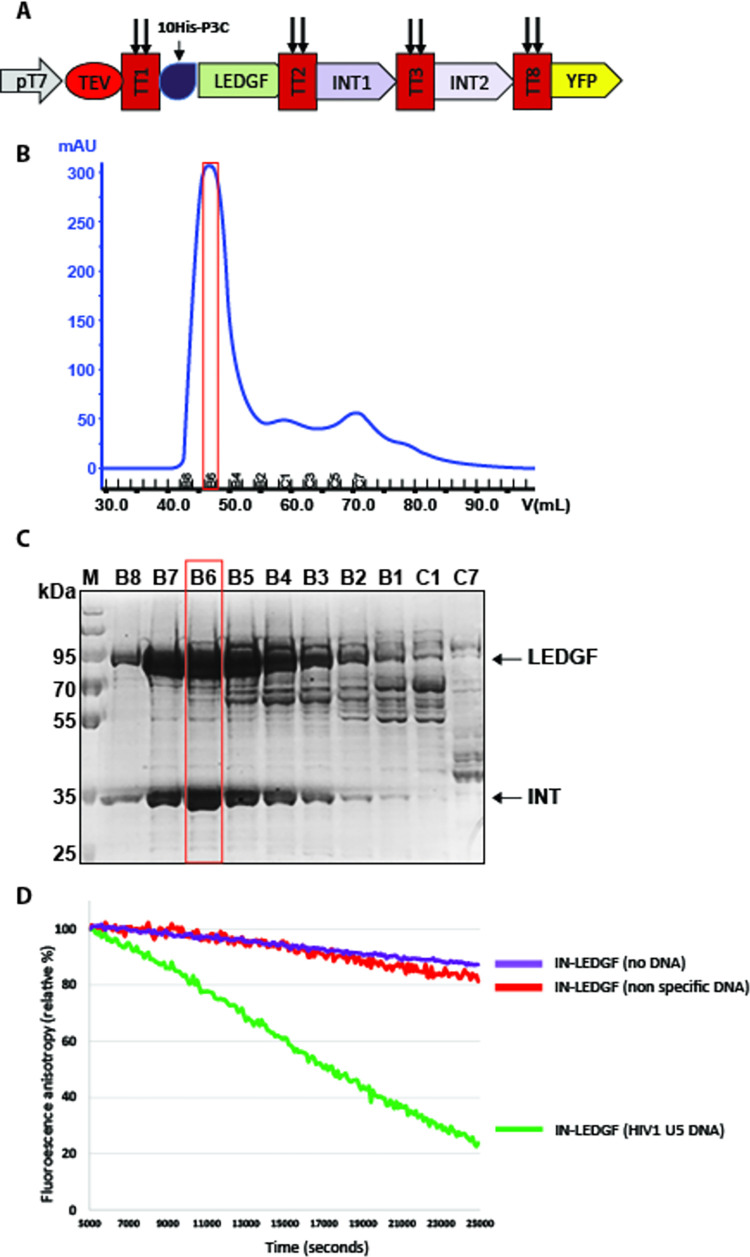
Expression of the IN/LEDGF complex. A. Schematic drawing of the ORFs inserted into the MVA-T7 virus behind a T7 promoter and encoding a polyprotein including from 5’ to 3’ the TEV protease, LEDGF, two distinct copies of the HIV-1 IN and YFP. Each ORF is separated from the other by twin TEV cleavage sites (perpendicular arrows). B. Purification of the IN-LEDGF complex from 12 10^9^ suspension BHK 21 cells infected with an MVA recombinant encoding the complex. The His-tagged complex was first purified by HPLC on a HisTrap excel Ni sepharose column (not shown) then the pooled fractions were concentrated and separated by size fractionation on a Hi Load 16/60 Superdex-200 column. The position of the B6 peak fraction is boxed in red. C. PAGE was performed on aliquots of the fractions recovered from the Superdex-200 column. Arrows point to the LEDGF and IN proteins stained with Coomassie blue. D. 3’ processing test of the IN/LEDGF complex. The release of GT-fluorescent dinucleotide was monitored by following fluorescence anisotropy as a function of time for the IN-LEDGF complex in the absence of DNA; in the presence of non-specific DNA or in the presence of HIV-1 U5 DNA.

**Fig 5:**
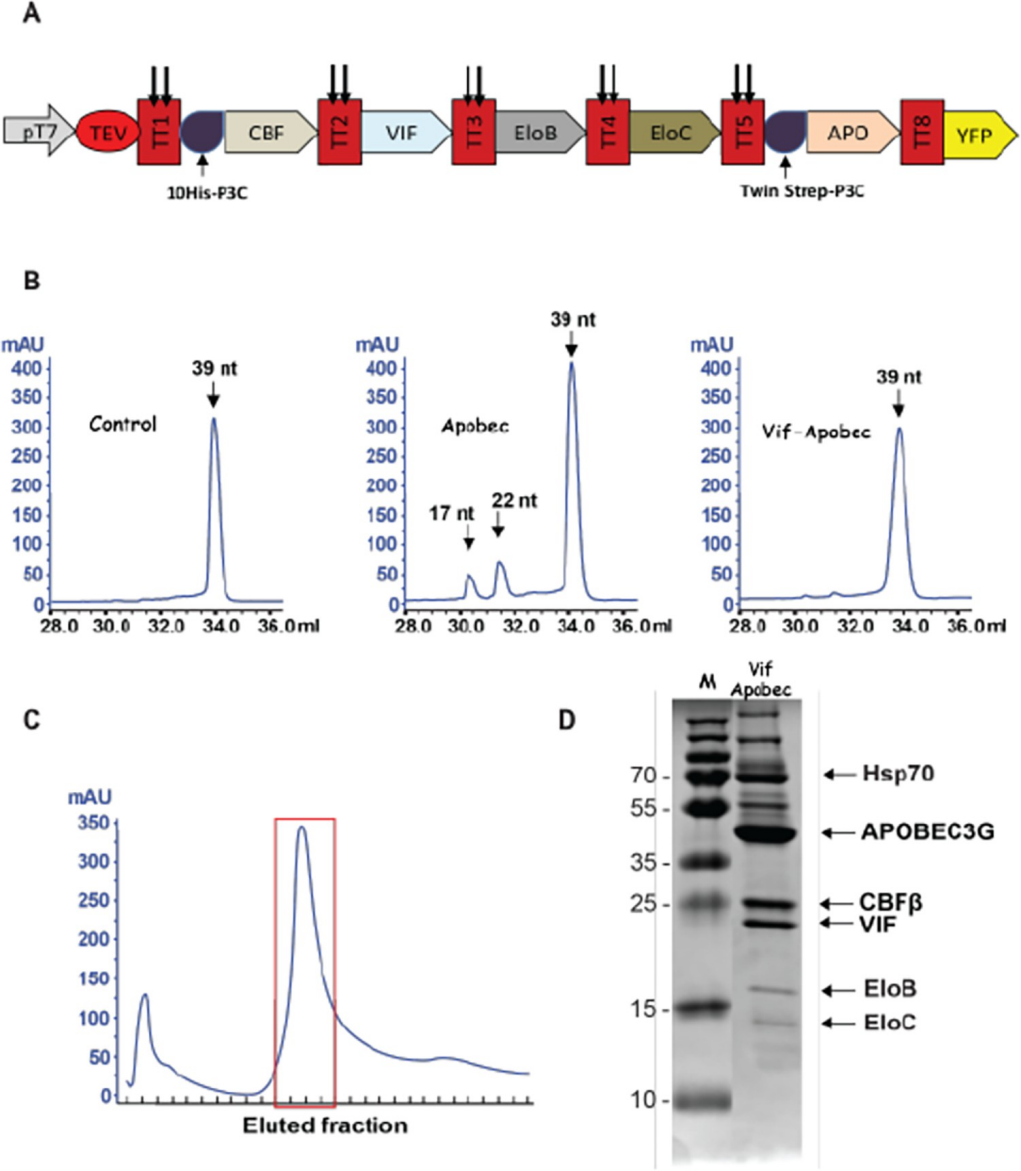
Expression of the Vif-APOBEC complex. A. Schematic drawing of the ORFs inserted into the MVA-T7 virus behind a T7 promoter and encoding a polyprotein including from 5’ to 3’ the TEV protease, CBFβ, VIF, EloB, EloC, APOBEC3G and YFP. Each ORF is separated from the other by twin TEV cleavage sites (perpendicular arrows). B. Cytidine deaminase activity in the absence of cell extract (left) or in the presence of cell extracts from BHK21 cells infected with MVA-T7 encoding APOBEC3G (middle) or MVA-T7 encoding the Vif-APOBEC complex (right). Enzymatic activity was determined in a two-step reaction where the appearance of the 17 and 22 oligonucleotide products was indicative of deaminase activity. C. Purification of the Vif-APOBEC complex from 2 liters of suspension BHK 21 C13-2P cells. Cell lysates clarified by ultracentrifugation at 100,000 g were injected onto a 1 ml StrepTactin® column and washed extensively. Peak fractions were combined, concentrated and examined by PAGE (right lane). Molecular weight markers are displayed in the left lane. Arrows point to proteins migrating with the mobility expected for APOBEC3G, CBFβ, Vif, EloB and Elo C. The identity of these proteins was confirmed by mass spectrometry. Proteins were stained with Coomassie blue.

In an initial attempt to express multiple proteins from a single virus recombinant, two genes (HIV 1 integrase and human lens epithelium-derived growth factor) were inserted into the MVA genome downstream of identical T7 promoters positioned at two distinct viral loci. Although both proteins were produced, only poor expression was obtained (not shown). We therefore chose an alternative strategy based on expression of cleavable polyproteins that have been successfully produced after expression in E. coli [17,18], in insect cells infected with baculovirus vectors [19,20] and in mammalian cells [21,22]. The genes encoding two or more proteins known to assemble into a complex were fused together into a single open reading frame downstream of an IPTG inducible T7 promoter. Each open reading frame, devoid of translation stop codons, was separated from the adjacent one by sequences encoding twin TEV cleavage sites (Figs 4A and 5A). Taking advantage of codon degeneracy, the protease cleavage site codons were designed to be unique at each ORF boundary to avoid recombination during virus propagation that could result in gene deletion. The gene encoding the TEV protease was inserted at the 5’ end of the construct immediately downstream of the T7 promoter so that the polyprotein would be cleaved upon expression and finally a gene encoding the yellow fluorescent protein (YFP) was inserted at the 3’ end to provide a readily visible marker of polyprotein expression. Gene assembly was performed from synthetic or PCR amplified ORFs using a BioBrick strategy [9,23] or Gibson cloning [24]. Expression plasmids were transfected into MVA-T7 infected cells and virus recombinants were selected with MPA and coumermycin counter-selection. In this manner two distinct protein complexes involved in replication of the human immunodeficiency virus HIV1 were overexpressed and purified.

The first protein complex, the HIV-1 preintegration complex, plays a key role in the integration of HIV cDNA into the host cell genome and is comprised of both HIV and host-cell encoded proteins. Two of the key protein players are the 32 kDa viral integrase (IN) and the 75 kDa host lens epithelium-derived growth factor (LEDGF) which tethers the preintegration complex to chromatin through its binding to IN. Further studies of this complex and its interaction with DNA are crucial for a full understanding of HIV infection. Our previous experiments on the IN-LEDGF complex have demonstrated that increased solubility and biochemical activity are observed when mammalian cells rather than E. coli or Baculovirus expression vectors are used to produce these proteins [6]. However, the process of producing and purifying individual proteins that are subsequently assembled into a complex is tedious. To express complexes preassembled in cells infected with a single recombinant virus, the genes encoding IN and LEDGF were fused together into one open reading frame (ORF) downstream of an IPTG inducible T7 promoter. Two copies of the gene encoding HIV-1 IN with distinct codons were introduced because previous studies established that the stoichiometry of the IN/LEDGF complex was two to one [25,26]. The 10His tag fused to the N-Terminus of LEDGF was used to purify the complex by affinity chromatography. After gel filtration (Fig 4B) and pooling fractions displaying the smallest amount of contaminating material by SDS-PAGE (4C), as much as 10 mg of the protein complex was obtained starting from 12 liters of suspension cells. The purified IN/LEDGF complex displayed characteristic 3’ processing activity on a DNA sequence that mimics the U5 end of HIV-1 cDNA but no activity in the presence of non-specific DNA (Fig 4D).

To further confirm the usefulness of the MVA-T7 platform for expression of a cleavable polyprotein, a complex involved in overcoming cell resistance to HIV infection was produced. The 23 kDa HIV-1 viral infectivity factor (VIF) targets the host restriction factor apolipoprotein B mRNA editing enzyme catalytic protein like (APOBEC) for degradation in the proteasome. For this to occur VIF enters into a complex in particular with the 46 kDa APOBEC3G protein and three cell proteins namely Core binding factor β (CBFβ, 22.2 kDa), Elongin B (EloB, 13.4 kDa) and Elongin C (EloC, 11 kDa), constituting part of the E3 ubiquitin ligase complex [27–29]. Thus, we constructed an MVA-T7 virus encoding the polyprotein CBFβ-VIF-EloB-EloC- APOBEC3G, referred to as the VIF-APOBEC complex (Fig 5A) as well as a control virus expressing only APOBEC3G. Whereas APOBEC3G, expressed on its own from the MVA-T7 genome displayed cytidine deaminase activity, expression of the VIF-APOBEC complex no longer allowed detection of such activity (Fig 5B) suggesting that association of APOBEC3G within the complex was inhibitory for its activity as expected from previous studies [30]. We then purified the Vif-APOBEC complex from 2 liters of infected cells (6x10^9^ cells) using the Twin-Strep tag at the N-terminus of APOBEC3G. Elution from the Strep-Tactin column allowed detection of a peak fraction where the most prominent Coomassie blue stained proteins corresponded in size to those predicted for APOBEC3G, CBFβ, Vif, EloB and EloC respectively (Fig 5C) while mass spectrometry confirmed their identity as well as their precise cleavage by the TEV protease. The complex was nevertheless contaminated with a few extraneous polypeptides, particularly Hsp70. Although further studies are necessary to determine whether a complex devoid of these additional species can be obtained, the results indicate that a protein complex composed of 5 different polypeptides can be assembled and purified from cells infected with an MVA-T7 recombinant virus using the polyprotein strategy.

## Discussion

The purpose of these studies has been to make the isolation of MVA recombinants faster and easier so as to facilitate the scale up of protein production in mammalian cells and enable the simultaneous synthesis of several polypeptides that interact to form a complex. There are several paths that can be taken to achieve such goals for instance the use of bacterial artificial chromosomes (BACs) containing the entire viral genome [31,32], CRISPR-Cas9 recombination [33,34] and even chemical synthesis of the entire MVA genome [35]. Whereas each of these methods has their own advantages and limitations we have found that the combination of positive selection for the GPT gene and negative selection against the GyrB-PKR gene with visual control of virus purification by following the loss of mCherry has considerably simplified the isolation of virus recombinants in our hands and resulted in consistent success. Negative coumermycin selection is attractive because coumermycin has no cytopathic effect at all over a two-day selection period. The ease of the combined selection system makes it feasible to isolate in parallel a number of virus recombinants simultaneously and we have done so for dozens of independent viruses. One could consider automating this system entirely to isolate hundreds of virus recombinants in one workflow. The overall time involved to have a recombinant virus stock for expression studies once a plasmid construct is available is 2 to 4 weeks but because tedious plaque isolation is not required, a limited amount of lab work is involved.

Production of large amounts of infected cells is critical for protein yields. The availability of a very fast growing BHK 21 suspension cell line (12 hour doubling time) and the establishment of a simple method for amplification and infection have allowed us so far to achieve as much as 10 mg yields of a protein complex from 12 liters of infected cell culture. Obviously, yields are highly dependent on the proteins being expressed, their solubility, stability and the efficiency of purification. We have focused particularly on the methods required to infect large cell volumes and found that adding virus to suspension cell cultures without any prior adsorption step in a reduced volume is simple and effective. We also report a simple way to infect large cell volumes by mixing uninfected cells with cells that have been previously infected at a low multiplicity. This considerably reduces the amount of virus required initially and saves time making virus stocks as well as storage space. A tradeoff is carrying out protein production over a 3-day period. For handling volumes beyond the 24 liters we have processed so far it could be useful to investigate other methods. The fact that cells are ultimately killed by the infection may appear to be an intrinsic limitation of the system however it is clearly feasible to grow cells to high densities for MVA production [36] as well as to produce MVA using continuous stirred tank procedures [37,38] raising the option of adapting these methods to industrial scale protein production with MVA vectors.

The ultimate aim, in our case to produce pure protein complexes for functional and structural studies, has been achieved by concatenating multiple ORFs into a single one, each being separated from the other by twin TEV cleavage sites, with the TEV protease being included in the polyprotein. This strategy has been inspired by prior work in other systems with the knowledge that alternative methods such as the use of picornavirus polyprotein processing may be equally suitable. In fact, Bahar et al. recently demonstrated the use of either a Foot and Mouse Disease Virus-2A sequence or a poliovirus internal ribosome entry site as alternative strategies to obtain individual proteins from a polyprotein sequence encoded by MVA [39]. However, these authors resorted to mixed infection of an MVA virus encoding the T7 RNA polymerase with an MVA virus encoding the polyprotein precursor. The method involving a single recombinant virus as reported here avoids the requirement to fine tune virus titers for mixed infections and thus facilitates volume scaleup.

Using the strategies developed in this report, a biochemically functional Integrase-LEDGF complex was produced in amounts sufficient for numerous enzymatic assays as well as structural studies while saving considerable time and effort. The alternative means involving the production of integrase and LEDGF on their own, then reconstituting the complex, has also been carried out but found to be considerably more time consuming. In addition, we have been successful in producing the Vif-APOBEC complex comprised of 5 distinct polypeptides although further studies will be required to improve the purity of the complex. Overall, the strategies described in this report should be valuable for the production of a variety of protein complexes difficult to produce in non- mammalian host systems and in many instances requiring post-translational modifications as well as a proper environment for folding.

## Supporting information

S1 Fig Construction of pKoom. (TIF)

S2 Fig DNA Sequences employed for ORF amplification and plasmid construction. (TIF)

S3 Fig Construction of plasmids for polyprotein expression from the MVA-T7 vector. (TIF)

S4 Fig Selection and amplification of MVA-T7 recombinants. (TIF)

S1 Table Expression of β glucuronidase in adherent BHK21 cells or suspension BHK21 C13-2P cells. (DOCX)

S1 Raw images (TIF)
