# Supplementary figures and images for: Efficient production of protein complexes in mammalian cells using a poxvirus vector
Source: PLoS One. 2022 Dec 15;17(12):e0279038. doi: 10.1371/journal.pone.0279038 (PMC9754296; doi:10.1371/journal.pone.0279038)

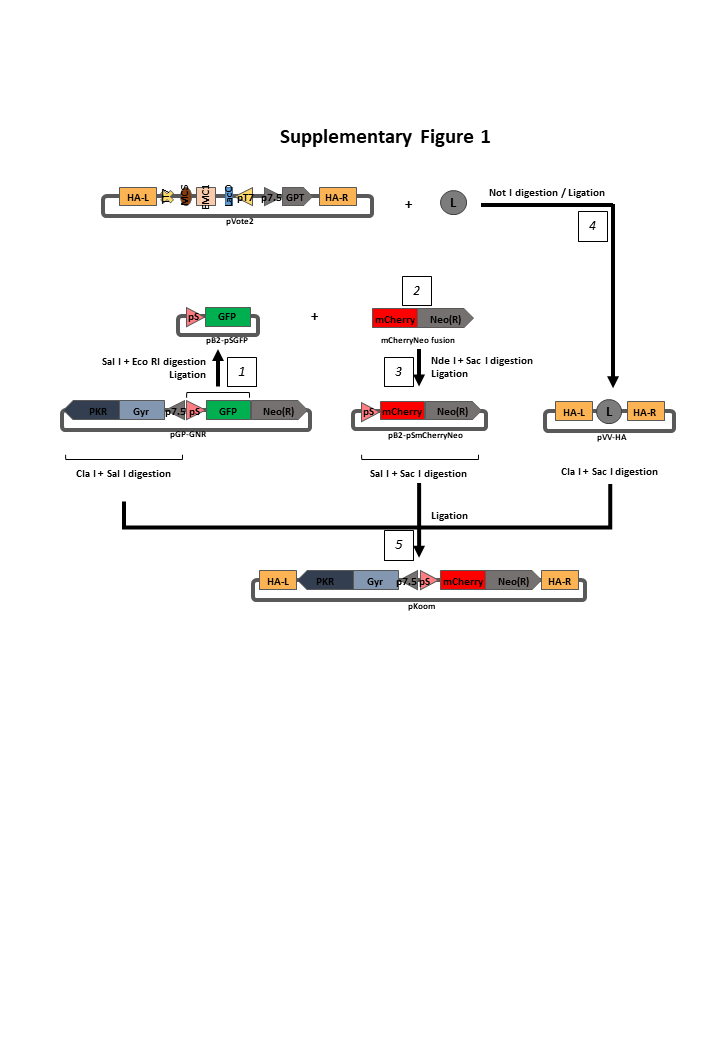

Supplement: S1 Fig — (TIF) [file pone.0279038.s001.tif]

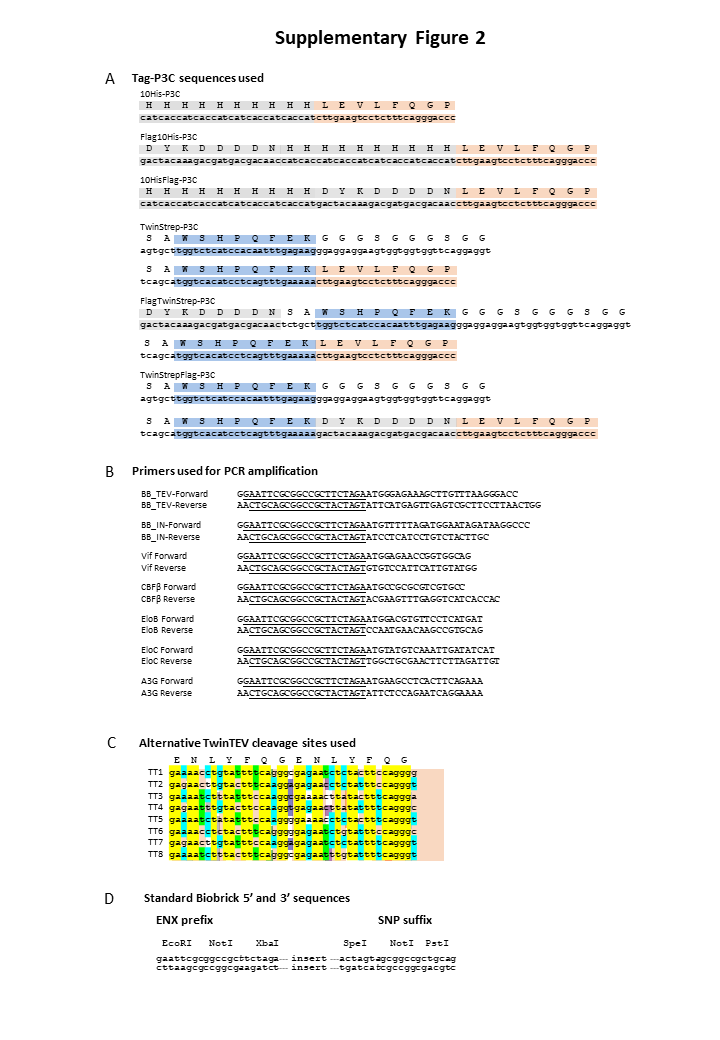

Supplement: S2 Fig — (TIF) [file pone.0279038.s002.tif]

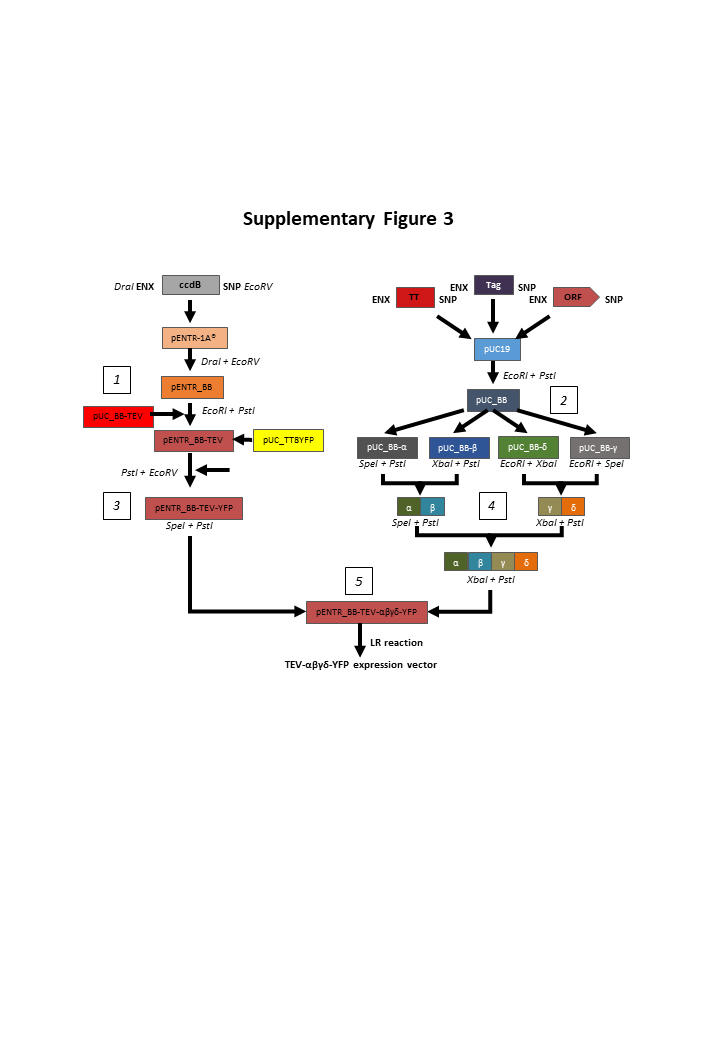

Supplement: S3 Fig — (TIF) [file pone.0279038.s003.tif]

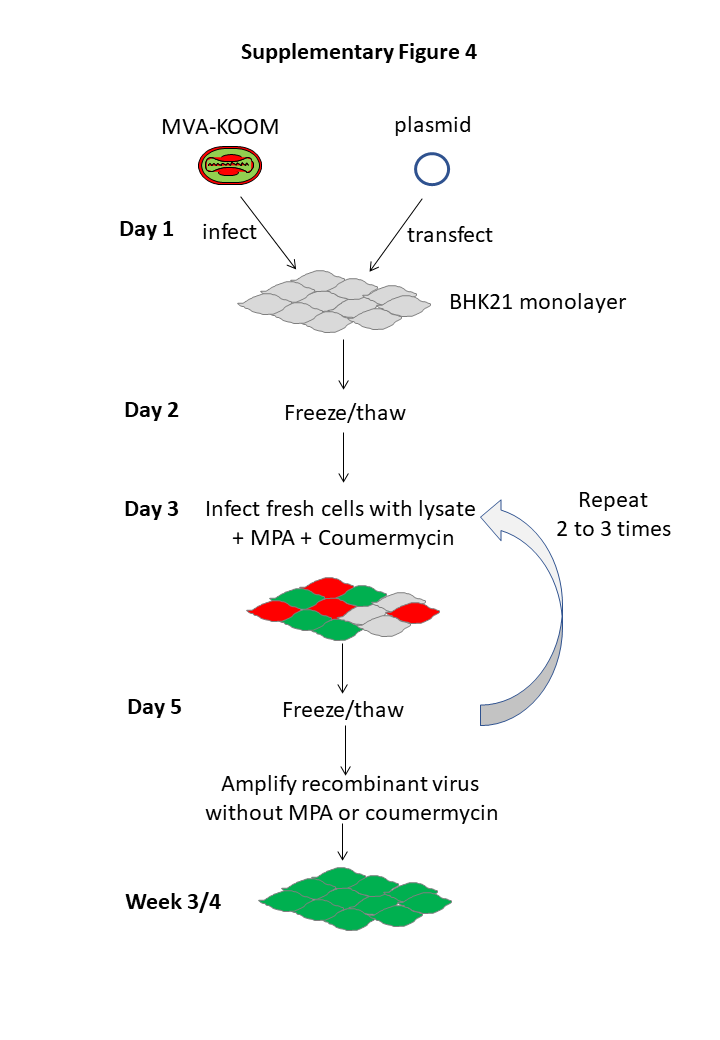

Supplement: S4 Fig — (TIF) [file pone.0279038.s004.tif]

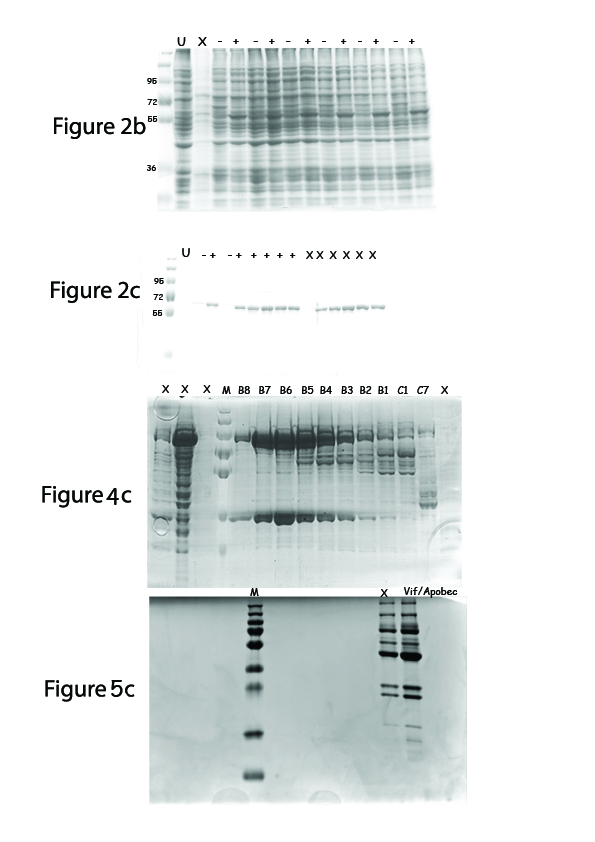

Supplement: S1 Raw images — (TIF) [file pone.0279038.s006.tif]
