# Supplementary material for: Efficient production of protein complexes in mammalian cells using a poxvirus vector
Source: PLoS One. 2022 Dec 15;17(12):e0279038. doi: 10.1371/journal.pone.0279038 (PMC9754296; doi:10.1371/journal.pone.0279038)
Supplement: S1 Table — (DOCX) [file pone.0279038.s005.docx]

**Supplementary table 1:**

**Expression of β glucuronidase in adherent BHK21 cells or suspension BHK21 C13-2P cells**

|  | Adherent cells | Suspension cells |
| --- | --- | --- |
| β glucuronidase | 54.5 ± 2.5 µg/10^6^ cells | 19.8 ± 0.4 µg/10^6^ cells |
